# Clinical characteristics and outcomes of cancer patients and their hospital course during the COVID-19 pandemic in a developing country

**DOI:** 10.1016/j.amsu.2022.103284

**Published:** 2022-01-30

**Authors:** Muhammad Sohaib Asghar, Farah Yasmin, Maryam Salma Babar, Muhammad Daim Bin Zafar, Syed Muhammad Ismail Shah, Muhammad Junaid Tahir

**Affiliations:** a Department of Internal Medicine at Dow University Hospital – Ojha Campus, Dow University of Health Sciences, Karachi, Pakistan; b Department of Internal Medicine, Dow Medical College, Dow University of Health Sciences, Karachi, Pakistan; c Department of Internal Medicine, Dubai Medical College, Dubai, United Arab Emirates; d Department of Internal Medicine at Ziauddin Medical University, Karachi, Pakistan; e Department of Internal Medicine, Lahore General Hospital, Lahore, Pakistan

**Keywords:** COVID-19, Cancer, Severity, Pandemic, Manifestations

## Abstract

In the new Coronavirus Disease 2019 (COVID-19) pandemic, cancer patients are considered a particularly susceptible population. We compared the type and magnitude of COVID-19 clinical manifestations among cancer patients in our center to non-cancer COVID-19 affected patients including 99 patients (28 cancer patients and 71 non-cancer patients). Hepatocellular carcinoma, breast carcinoma, and leukemia were the most common cancers. Diabetes and hypertension were prevalent comorbidities. Dyspnea, cough, fatigue, myalgia and diarrhea were statistically indifferent in both groups. Fatigue was more pronounced in cancer patients [OR: 2.573(1.025–6.460), p = 0.041] along with early onset of bilateral patchy consolidation [HR: 3.127(1.197–5.851), p = 0.032].

## Funding sources

No funding required for the study.

## Ethical approval statement

Ethical approval was taken in this study from institutional review board of Dow University Hospital, Karachi (IRB/DUH/2021/742021), and consent to participate was not required due to retrospective nature of the study.

## Data availability statement

All data will be made available on a reasonable request to the corresponding author.

## Provenance and peer review

Not commissioned, externally peer reviewed.

## Registration of research study

Registration of study protocol was done in Ethical Review Committee of Dow University Hospital (IRB/DUH/2021/742021) prior to the commencement of the study.

In the new Coronavirus Disease 2019 (COVID-19) pandemic, cancer patients are considered a particularly susceptible population. To date, little is known about the clinical features of COVID-19-infected cancer patients. We compare the type and magnitude of COVID-19 clinical manifestations among cancer patients in our center to non-cancer COVID-19 affected patients. A retrospective study was conducted between March and October 2020, for a duration of 7 months at a tertiary care hospital. There were 99 patients evaluated during their COVID-19 disease course. This included 28 cancer patients and 71 non-cancer patients. We compared the type and magnitude of COVID-19 clinical manifestations among cancer patients in our center to non-cancer COVID-19 affected patients. The study was conducted according to Declaration of Helsinki after waiver from ethical review board. All analysis was conducted using SPSS version 25.0 (IBM Corp, Armonk, NY) and variables were reported using descriptive statistics. Descriptive continuous variables were calculated by independent *t*-test, while qualitative analysis was done using either chi-square test or Fisher's exact test as indicated.

**Fig. 1:**
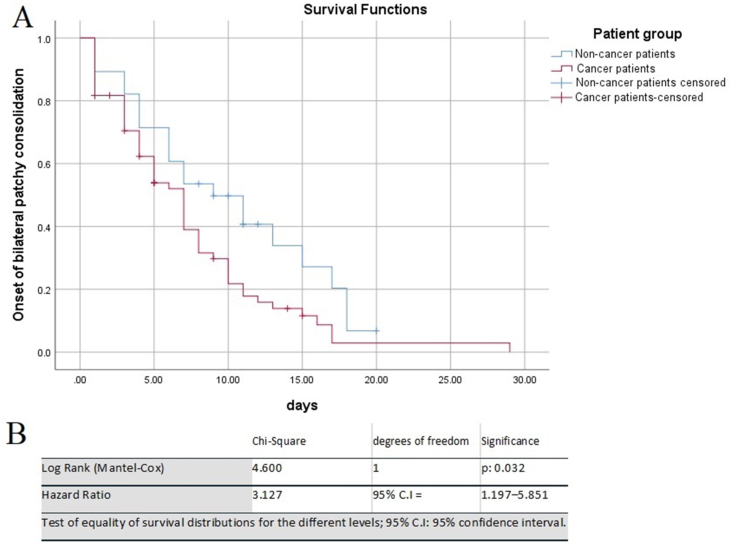
Kaplan-Meier curve showing onset of bilateral patchy consolidation (A) on days after admission in cancer and non-cancer patients. (B) Inferential statistics showing significant hazard ratio 3.127 (1.197–5.851) with log-rank test (p = 0.032).

The average age of the cancer patients in our study was 57.32 years, with 64.3% of them comprising men. As compared to the non-cancer population, cancer patients were considerably older (p < 0.001). In our cohort, hepatocellular carcinoma, breast carcinoma, and leukemia were the most common cancers. Around 32.1% of cancer patients were in stage I of their disease. Mode of therapy for majority (46.4%) of these patients was surgery while a few patients were treated with chemotherapy alone, radiotherapy alone, or both. Majority (78.6%) of the cancer patients from this cohort were not currently using chemotherapy or radiotherapy. Diabetes and hypertension were found to be slightly more prevalent (43.6% and 38.0%) in non-cancer patients compared to cancer patients. However, IHD, CVA, CLD and COPD/Asthma were insignificantly more prevalent in cancer patients. Symptoms such as dyspnea, cough, fatigue, myalgia, diarrhea were more common in the cancer patients (statistically insignificant). Fatigue was more pronounced in cancer patients than in the non-cancer population, with an odds ratio of 2.573 and a confidence interval of 1.025–6.460 (p = 0.041). On Kaplan-Meier curve, early onset of bilateral patchy consolidation was evident in cancer patients (p = 0.032) with a hazard ratio of 3.127 and a confidence interval of 1.197–5.851 as shown in Fig. 1. Table 1 and Table 2 includes the baseline characteristics of cancer patients, their comparison with non-cancer patients, and in-hospital events and laboratory data of the study population.

**Table 1:** Baseline clinical characteristics of COVID-19 infected cancer patients (n = 28).

Mean age (in years)	57.32 (13.28)
Gender	Males: 18 (64.3%)
	Females: 10 (35.7%)
Comorbidities	Diabetes: 11 (39.3%)
	Hypertension: 5 (17.6%)
	Cardiovascular disease: 4 (14.3%)
	Cerebrovascular disease: 2 (7.1%)
	Chronic pulmonary disease: 3 (10.7%)
	Chronic liver disease: 5 (17.6%)
	Chronic kidney disease: 1 (3.6%)
Tumor diagnosis	Hepatocellular carcinoma: 5 (17.6%)
	Lung carcinoma: 2 (7.1%)
	Breast carcinoma: 5 (17.6%)
	Prostatic carcinoma: 1 (3.6%)
	Squamous cell carcinoma of oral cavity: 1 (3.6%)
	Endometrial carcinoma: 1 (3.6%)
	Ovarian carcinoma: 1 (3.6%)
	Leukemia: 4 (14.3%)
	Lymphoma: 1 (3.6%)
	Colonic carcinoma: 2 (7.1%)
	Rectal carcinoma: 3 (10.7%)
	Esophageal carcinoma: 1 (3.6%)
	Renal cell carcinoma: 1 (3.6%)
Tumor staging	Stage I: 9 (32.1%)
	Stage II: 8 (28.6%)
	Stage III: 4 (14.3%)
	Stage IV: 7 (25.0%)
Mode of therapy	Surgery: 13 (46.4%)
	Chemotherapy alone: 5 (17.6%)
	Radiotherapy alone: 4 (14.3%)
	Combined chemo/radio therapy: 6 (21.4%)
Current use of chemo/radio therapy	Yes: 6 (21.4%)
	No: 22 (78.6%)
Medical therapy given for COVID-19	Antibiotics: 21 (75.0%)
	Lopinavir/ritonavir: 2 (7.1%)
	Corticosteroids: 23 (82.1%)
	Vitamin supplements: 25 (89.3%)
	Tocilizumab: 1 (3.6%)
	Remdesivir: 8 (28.6%)
	Hydroxychloroquine: 5 (17.6%)
	Anticoagulant therapy: 14 (50.0%)

Data presented as either frequency (%), or mean and standard deviation (SD).

**Table 2:** Comparison of baseline data of the study population (A), hospital stay and laboratory markers (B) among the cancer and non-cancer patients (n = 99).

(A) Variables	Cancer patients (n = 28)	Non-cancer patients (n = 71)	OR (95% Confidence Interval)	P-value	
Median Age	60 (52.25–67.75)	44 (35.00–53.00)	–	**<0.001**^a^	
Gender	Males: 60.0% (n = 18) Females: 40.0% (n = 10)	Males: 53.5% (n = 38) Females: 46.5% (n = 33)	1.563 (0.634–3.856)	0.332^b^	
BMI (kg/m^2^)	25.40 (19.40–27.00)	26.20 (24.20–28.40)	–	0.198^a^	
Baseline creatinine (mg/dL)	1.22 (0.34)	1.13 (0.40)	–	0.297^c^	
Diabetes	39.3% (n = 11)	43.6% (n = 31)	0.835 (0.342–2.037)	0.692^b^	
Hypertension	17.6% (n = 5)	38.0% (n = 27)	0.354 (0.120–1.042)	0.060^d^	
IHD	14.3% (n = 4)	7.0% (n = 5)	2.200 (0.545–8.880)	0.266^d^	
CVA	7.1% (n = 2)	4.2% (n = 3)	1.744 (0.275–11.038)	0.620^d^	
CLD	17.9% (n = 5)	5.6% (n = 4)	3.641 (0.900–14.729)	0.112^d^	
ILD/COPD/Asthma	10.7% (n = 3)	8.5% (n = 6)	1.300 (0.302–5.601)	0.725^d^	
Chronic kidney disease	3.6% (n = 1)	4.2% (n = 3)	0.840 (0.084–8.429)	0.882^d^	
Fever	78.6% (n = 22)	85.9% (n = 61)	0.601 (0.195–1.848)	0.377^d^	
Dyspnea	71.4% (n = 20)	70.4% (n = 50)	1.050 (0.400–2.757)	0.921^b^	
Cough	82.1% (n = 23)	71.8% (n = 51)	1.804 (0.602–5.401)	0.292^b^	
Fatigue	67.9% (n = 19)	47.9% (n = 34)	2.573 (1.025–6.460)	**0.041**^b^	
Myalgia	46.4% (n = 13)	39.4% (n = 28)	1.331 (0.551–3.216)	0.525^b^	
Diarrhea	25.0% (n = 7)	18.3% (n = 13)	1.487 (0.523–4.231)	0.455^b^	
ARDS	17.9% (n = 5)	9.9% (n = 7)	1.988 (0.574–6.886)	0.312^d^	
Mortality	25.0% (n = 7)	15.5% (n = 11)	1.818 (0.624–5.301)	0.269^b^	
Invasive ventilation	14.3% (n = 4)	12.7% (n = 9)	1.148 (0.323–4.082)	0.831^d^	
Recovery	75.0% (n = 21)	84.5% (n = 60)	0.550 (0.189–1.603)	0.273^b^	
**(B) Variables**		**Cancer patients (n = 28)**	**Non-cancer patients (n = 71)**	**p-value**	
Hospital stay					
Length of stay (in days)		8.50 (4.00–11.75)	5.00 (1.00–8.00)	**0.010**^a^	
Duration of fever (in days)		5.32 (3.28)	4.95 (2.170)	0.514^c^	
Onset of bilateral patchy consolidation (in days)		7.11 (4.52)	9.55 (3.99)	**0.016**^c^	
Onset of dyspnea after illness (in days)		4.00 (3.00–5.00)	6.00 (4.00–9.43)	**0.006**^a^	
Onset of ARDS (in days)		7.92 (2.79)	7.14 (3.08)	0.663^c^	
Time from diagnosis to death (in days)		9.74 (4.12)	11.37 (3.69)	0.395^c^	
Time from onset of symptoms to hospitalization (in days)		3.42 (1.15)	5.54 (1.65)	**<0.001**^c^	
Time from onset of dyspnea to mechanical ventilation (in days)		2.14 (2.04)	2.99 (1.58)	0.196^c^	
Hematological markers					
Leukocytes ( × 10^9^per L)		10.70 (6.90–14.60)	9.60 (6.80–12.50)		0.288^a^
Hemoglobin (g/L)		11.69 (2.42)	11.43 (2.05)		0.592^c^
Lymphocytes (%)		10.00 (7.00–26.00)	23.00 (16.00–28.00)		**0.004**^a^
Neutrophils (%)		77.25 (12.57)	71.36 (11.17)		**0.039**^c^
Platelets ( × 10^9^per L)		244.00 (186.00–340.00)	225.50 (160.75–302.75)		0.361^a^
Mean corpuscular volume (fL)		85.00 (80.00–89.00)	84.72 (76.00–88.93)		0.495^c^
Neutrophil to lymphocyte ratio (NLR)		8.60 (2.35–12.57)	3.13 (2.35–5.00)		**0.008**^a^
Lymphocyte to monocyte ratio (LMR)		2.66 (1.20–4.33)	4.25 (3.00–6.00)		**0.001**^a^
Platelet to lymphocyte ratio (PLR)		199.48 (127.34–285.28)	147.55 (237.75)		0.233^a^
Lymphocyte to CRP ratio (LCR)		90.90 (34.67–571.98)	177.07 (65.85–1059.37)		0.094^a^
Biochemistry panel					
Urea (mg/dL)		30.06 (18.67–48.25)	23.75 (18.09–39.50)		0.235^a^
Creatinine on admission (mg/dL)		0.83 (0.75–1.20)	0.72 (0.63–0.95)		0.011^a^
Sodium (mEq/L)		137.50 (136.00–140.75)	140.00 (138.00–143.00)		**0.003**^a^
Potassium (mEq/L)		4.10 (3.80–4.37)	3.90 (3.60–4.40)		0.115^a^
Chloride (mEq/L)		102.00 (98.00–105.00)	105.50 (103.00–108.00)		**0.001**^a^
Bicarbonate (mEq/L)		19.07 (4.23)	20.67 (3.55)		0.062^c^
Inflammatory biomarkers					
C-reactive protein (mg/L)		116.63 (35.00–274.30)	54.60 (15.65–172.10)		**0.049**^a^
Ferritin (ng/mL)		760.29 (195.67–2000.00)	319.23 (118.08–949.75)		0.133^a^
Procalcitonin (ng/ml)		0.61 (0.03–3.02)	0.20 (0.11–0.68)		0.641^a^
Lactate dehydrogenase (U/L)		568.00 (348.50–707.00)	468.00 (353.00–660.00)		0.740^a^
D-dimer (mcg/mL)		1.92 (1.53–2.49)	2.42 (0.84–9.84)		0.705^a^
Liver function enzymes					
ALT (U/L)		30.50 (20.50–48.25)	34.00 (24.00–50.00)		0.436^a^
AST (U/L)		40.00 (26.75–58.00)	41.00 (30.00–54.00)		0.861^a^
Total bilirubin (mg/dL)		0.56 (0.28–0.85)	0.48 (0.32–0.76)		0.509^a^
Direct bilirubin (mg/dL)		0.35 (0.14–0.45)	0.25 (0.16–0.43)		0.323a
Indirect bilirubin (mg/dL)		0.25 (0.13–0.36)	0.22 (0.15–0.32)		0.569^a^
Gamma glutamyl transferase (U/L)		84.50 (45.75–147.75)	49.00 (28.00–80.00)		**0.016**^a^
Alkaline phosphatase (U/L)		94.50 (75.00–134.50)	89.00 (64.00–114.00)		0.267^a^

Abbreviations: IHD: ischemic heart disease; CVA: cerebrovascular accident; COPD: chronic obstructive pulmonary disease; ARDS: acute respiratory distress syndrome; CKD: chronic kidney disease; OR: odds ratio; ILD: interstitial lung disease; BMI: body mass index; COVID-19: coronavirus disease 2019; ALT, Alanine aminotransferase; AST, Aspartate aminotransferase; CRP, C-reactive protein.

a Indicates p-value calculated by Mann Whitney *U* test.

b Indicates chi-square test.

c Indicates student's t-test.

d Indicates Fisher's exact test (Test of significance is determined after checking normality of the data through Shapiro-Wilk test).

We shed light on clinical manifestations seen in cancer patients infected with the novel Corona Virus 2019 (COVID-19) in a report conducted by Zhang et al. [1] in Wuhan, China. As per their findings, lung cancer was prevalent among COVID-19 patients, who had higher rates of hospital-acquired transmissions. Lymphopenia, elevated C-reactive protein (CRP), anemia, and hypoproteinemia were all shown to be major factors in the disease's progression. Fever, cough, and dyspnea were the most common clinical symptoms observed, while ground-glass opacities and patchy consolidations were common CT-scan findings. Patients undergoing anti-cancer treatment within 14 days had a higher risk of mortality, and patients with patchy convergence on CT-scan had a higher risk of adverse effects. The overall mortality rate was reported to be 28.6%. Cancer patients with COVID-19 infection have a poor prognosis and performance, and immunocompromised people have a greater risk of infection.

Owing to their immunocompromised conditions, patients with malignancies are more likely to acquire infection and are at a greater risk of contracting critical illness and elevated mortality as a result of COVID-19 [2]. The median age of COVID-19-infected cancer patients is 65–66 years (66–80), with males being the most affected in multiple trials [1,[3], [4], [5]]. As compared to the non-cancer population, cancer patients were considerably older in our study. Lung cancer was shown to be more frequent in COVID-19-positive cancer patients in multiple studies [1,4,[6], [7], [8]]. However, in our cohort, hepatocellular carcinoma, breast carcinoma, and leukemia were the most common cancers. In comparison to diabetes, hypertension was a prominently reported comorbidity in multiple trials (39.3%) [1,2,[7], [8], [9], [10]]. Numerous trials [1,8] found that the majority of patients contracted the disease from a hospital-acquired route of transmission. The majority of research [2,3,5] found that patients experienced fever, dry cough, nausea, and dyspnea as distinct clinical symptoms. Both of these signs were normal in our sample, but fatigue was more pronounced in cancer patients. Leukocytosis, lymphopenia, elevated levels of C-reactive protein (CRP), procalcitonin (PCT), D-dimer, IL-6 (Interleukin-6), Neutrophil to Lymphocyte ratio (NLR), and Lactate dehydrogenase (LDH) have all been observed in COVID-19-infected cancer patients [[1], [2], [3],^7^]. When compared to other studies [1], Zhang et al. recorded normal levels of procalcitonin along with elevated levels of erythrocyte sedimentation rate (ESR) and globulins, as well as a decrease in albumin levels coinciding with a trivial sample [2]. Certain biochemical indicators such as neutrophilia (p = 0.039), lymphocytopenia (p = 0.009), increased NLR (p = 0.008), decreased LMR (p = 0.001), decreased serum sodium (p = 0.003), chloride levels (p = 0.001), increased gamma glutamyl transferase phosphatase (p = 0.014), CRP (p = 0.049), were substantially significant in cancer patients when compared to non-cancer patients. Patchy consolidations and ground-glass opacities were typical radiological (CT-scan) findings in COVID-19-infected cancer patients [1,7]. On the other hand, Yang et al. [2] cited bilateral inflammatory infiltrates as a prominent observation. ARDS (acute respiratory distress syndrome) was a common complication reported in several reports [1,2,7]. On the Kaplan-Meier curve, our cancer patients had early-onset bilateral patchy infiltrates as compared to non-cancer patients (p = 0.032). In trivial trials, the average length of stay in the hospital was 16–19 days [1,2,10]. Cancer patients had a longer hospital stay (p = 0.023), early development of dyspnea (p = 0.001), and early hospitalization from the onset of symptoms (p = 0.001) than non-cancer patients in our research. Few studies [1,7] show that non-invasive breathing and oxygen therapy are commonly used by management. Different studies have shown that cancer patients exposed to COVID-19 have a mortality rate of 25–28% [1,3,4,9]. Increased age, the involvement of two or more comorbidities, deranged hematological parameters, elevated CRP, PCT, NLR, D-dimer, LDH levels, irregular CT-scan results, and palliative therapy are all linked to the deteriorating condition in COVID-19 patients, potentially increasing the risk of death [3,5,7,9,10]. In conclusion, we found no major differences in mortality, invasive breathing, or the prevalence of ARDS between cancer and non-cancer patients.

## Declaration of competing interest

The authors have no conflicts of interest to declare.

## References

[1] Zhang L., Zhu F., Xie L., Wang C., Wang J., Chen R. (2020). Clinical Characteristics of COVID-19-infected cancer patients: a retrospective case study in three hospitals within Wuhan, China. Ann. Oncol..

[2] Yang K., Sheng Y., Huang C., Jin Y., Xiong N., Jiang K. (2020). Clinical characteristics, outcomes, and risk factors for mortality in patients with cancer and COVID-19 in Hubei, China: a multicenter, retrospective, cohort study. Lancet Oncol..

[3] Lui G., Ling L., Lai C.K.C. (2020). Clinical characteristics and prognosis in cancer patients with COVID-19: a single center's retrospective study. Letter to the Editor. J. Infect..

[4] Hsu S.H., Wang S.Y. (2020). SARS-CoV-2 transmission in patients with cancer at a tertiary care hospital in Wuhan, China. JAMA Oncol..

[5] Kuderer N.M., Choueir T.K., Shah D.P., Shyr Y., Rubinstein S.M., Rivera D.R. (2020). Clinical impact of COVID-19 on patients with cancer (CCC19): a cohort study. Lancet.

[6] Liang W., Guan W., Chen R., Wang W., Li J., Xu K. (2020). Cancer patients in SARS-CoV-2 infection: a nationwide analysis in China. Lancet.

[7] Yang F., Shi S., Zhu J., Shi J., Dai K., Chen X. (2020). Clinical characteristics and outcomes of cancer patients with COVID-19. J. Med. Virol..

[8] Dai M., Liu D., Liu M., Zhou F., Li G., Chen Z. (2020). Patients with cancer appear more vulnerable to SARS-CoV-2: a multicenter study during the COVID-19 outbreak. Am. Assoc. Cancer. Res..

[9] Lee L.Y.W., Cazier J.B., Angelis V., Arnold R., Bisht V., Campton N.A. (2020). COVID-19 mortality in patients with cancer on chemotherapy or other anticancer treatments: a prospective cohort study. Lancet.

[10] Abbattista M., Ciavarella A., Capecchi M., Tantardini F., Gramegna A., Scaramellini N. (2020). Risk factors for mortality in hospitalized patients with COVID-19: a study in Milan, Italy. Inf. Disp..

